# A Simplified Model of Communication Between Time Cells: Accounting for the Linearly Increasing Timing Imprecision

**DOI:** 10.3389/fncom.2018.00111

**Published:** 2019-01-29

**Authors:** Mustafa Zeki, Fuat Balcı

**Affiliations:** ^1^Department of Mathematics, College of Engineering and Technology, American University of the Middle East, Egaila, Kuwait; ^2^Department of Psychology, Koç University, Istanbul, Turkey

**Keywords:** interval timing, scalar variability, time cells, chain models, hippocampus, Weber's law

## Abstract

Many organisms can time intervals flexibly on average with high accuracy but substantial variability between the trials. One of the core psychophysical features of interval timing functions relates to the signatures of this timing variability; for a given individual, the standard deviation of timed responses/time estimates is nearly proportional to their central tendency (scalar property). Many studies have aimed at elucidating the neural basis of interval timing based on the neurocomputational principles in a fashion that would explain the scalar property. Recent experimental evidence shows that there is indeed a specialized neural system for timekeeping. This system, referred to as the “time cells,” is composed of a group of neurons that fire sequentially as a function of elapsed time. Importantly, the time interval between consecutively firing time cell ensembles has been shown to increase with more elapsed time. However, when the subjective time is calculated by adding the distributions of time intervals between these sequentially firing time cell ensembles, the standard deviation would be compressed by the square root function. In light of this information the question becomes, “How should the signaling between the sequentially firing time cell ensembles be for the resulting variability to increase linearly with time as required by the scalar property?” We developed a simplified model of time cells that offers a mechanism for the synaptic communication of the sequentially firing neurons to address this ubiquitous property of interval timing. The model is composed of a single layer of time cells formulated in the form of integrate-and-fire neurons with feed-forward excitatory connections. The resulting behavior is simple neural wave activity. When this model is simulated with noisy conductances, the standard deviation of the time cell spike times increases proportionally to the mean of the spike-times. We demonstrate that this statistical property of the model outcomes is robustly observed even when the values of the key model parameters are varied.

## 1. Introduction

Time is a fundamental quantity that cannot be derived from other dimensions. Thus, keeping track of time requires its measurement by a neural “clock” mechanism. To that end, evolution has favored at least two timing mechanisms that operate at different time-scales. One of these timekeeping mechanisms, namely the circadian clock, captures periods with 24-h long cycles based on well-regulated molecular machinery (e.g., Partch et al., 2014). Many events in nature, on the other hand, are rarely periodic and/or too short to be captured based on the time-scales of the molecular events implicated for the circadian clock. Thus, capturing the temporal features of such events requires a rather flexible timekeeping apparatus that can be started and stopped arbitrarily, namely a neural mechanism with stopwatch-like properties (Buhusi and Meck, 2005). Accordingly, a mechanism of the latter type indeed enables many animals ranging from fish (Drew et al., 2005), to mice (Balci et al., 2009), to humans (Rakitin et al., 1998; Çavdaroğlu et al., 2014) to flexibly keep track of time intervals in the range of seconds to minutes. This very ability is referred to as “interval timing.”

There are a number of core features of this ubiquitous cognitive timekeeping function. For instance, for a given target time interval, the timed anticipatory responses of animals are approximately Gaussian distributed, which is typically centered at around the target interval, pointing at on-average high timing accuracy. However, the flexibility of this mechanism exerts a non-negligible cost in the form of imprecision: predictions/productions of a given target interval exhibit substantial variability between trials, which is reflected in the spread of the response time distributions. Thus, when it comes to precision, the operation of the internal stopwatch is far from perfect (i.e., outputs are not Dirac delta distributed). Importantly, within an individual, the resultant timing imprecision has a well-defined relationship to the target intervals; the standard deviation of the timed responses is proportional to their mean, namely the coefficient of variation of timed responses is virtually constant. This statistical property leads to the timescale invariance of interval timing and accounts for Weber's Law in the timing domain (Gibbon, 1977).

A line of empirical and theoretical research in the timing field has focused on the neurocomputational principles that would explain interval timing with its psychophysical features outlined above (for review see Karmarkar and Buonomano, 2007; Simen et al., 2011; Merchant et al., 2013; Hass and Durstewitz, 2014; Balcı and Simen, 2016). A recent line of neuroscientific evidence has introduced novel empirical ground for these approaches by demonstrating the existence of a specialized neural mechanism for timekeeping, namely the time cell ensembles that fire sequentially during different episodes of a temporally structured task (Kraus et al., 2013; Salz et al., 2016; Tiganj et al., 2016). Importantly, as a feature of information processing in the time cell architecture, the time interval from the activation of one ensemble to the next ensemble (inter-spike interval, ISI) has been shown to lengthen with progressing neuronal activity (MacDonald et al., 2011). In a very simple network of a neural chain architecture with feedforward excitatory connections, we aimed to address how the communication between the consecutive cell ensembles can be set to achieve scalar variability of interval timing behavior.

Even in very simplified cell and network settings, one needs to address several neurocomputational challenges to explain the statistical features of experimentally observed activity/behavior as a function of time. One of these features is the scalar property of interval timing. The challenge faced here is that when the distributions are added together, the standard deviation of the resulting distribution is compressed by the square root function. For example, when *N* identical normal distributions with mean = 1, 000 and the standard deviation = 100 are summed, the mean of the resulting distribution would be 1, 000*N* and its standard deviation would be 100*N*. Thus, the standard deviation would not increase linearly with the mean, contrary to what would be required by the scalar property. The important question that arises at this point is whether it is possible to compensate for the compression in variability (i.e., CV) due to the square root function via some inherent property of neural networks and thereby explain the scalar property as an emergent property of the network.

The second challenge faced in accounting for the experimentally observed activity with realistic neurons is the limiting timescale of the neuronal currents. Whether intrinsic or synaptic, many known neuronal currents operate in a timescale ranging from milliseconds to a few seconds. But the increase of ISIs in time cells (TC) is observed for as long as tens of seconds (Ermentrout and Terman, 2010). This motivated us to ask how neuronal currents with very short lifetimes can be used to generate effects that last much longer than their individual timescales.

In order to address such neurocomputational issues in signal transmission between the time cells, we constructed a simple time cell model in such a way that the delay from the firing of the *i*_*th*_ time cell ensemble to the firing of the *i* + 1_*th*_ ensemble increases with the inhibition. This is due to the hyperpolarizing intrinsic current that is activated with inhibition and inactivated with excitation. In other words, in this model, the time cells undergo a temporal integration that depends on the level of inhibitory current. In the chain architecture, when the time cells are connected with feedforward excitatory current, the way the time cells are modeled leads to experimentally observed increasing ISIs with propagating activity in the chain. We simulated the outlined network with noisy conductances multiple times to generate the distribution of spike times of various time cells. We observed that the standard deviation of the time cell spike times indeed increased linearly with the mean spike times and the mean-normalized distributions of different time cell activity superposed as often observed in the empirical data (i.e., time-scale invariance). We finally showed that the observed results are the robust features of the model outputs that are preserved even after changing the values of the key parameters of the model.

## 2. Methods: The Model

**Figure 1:**
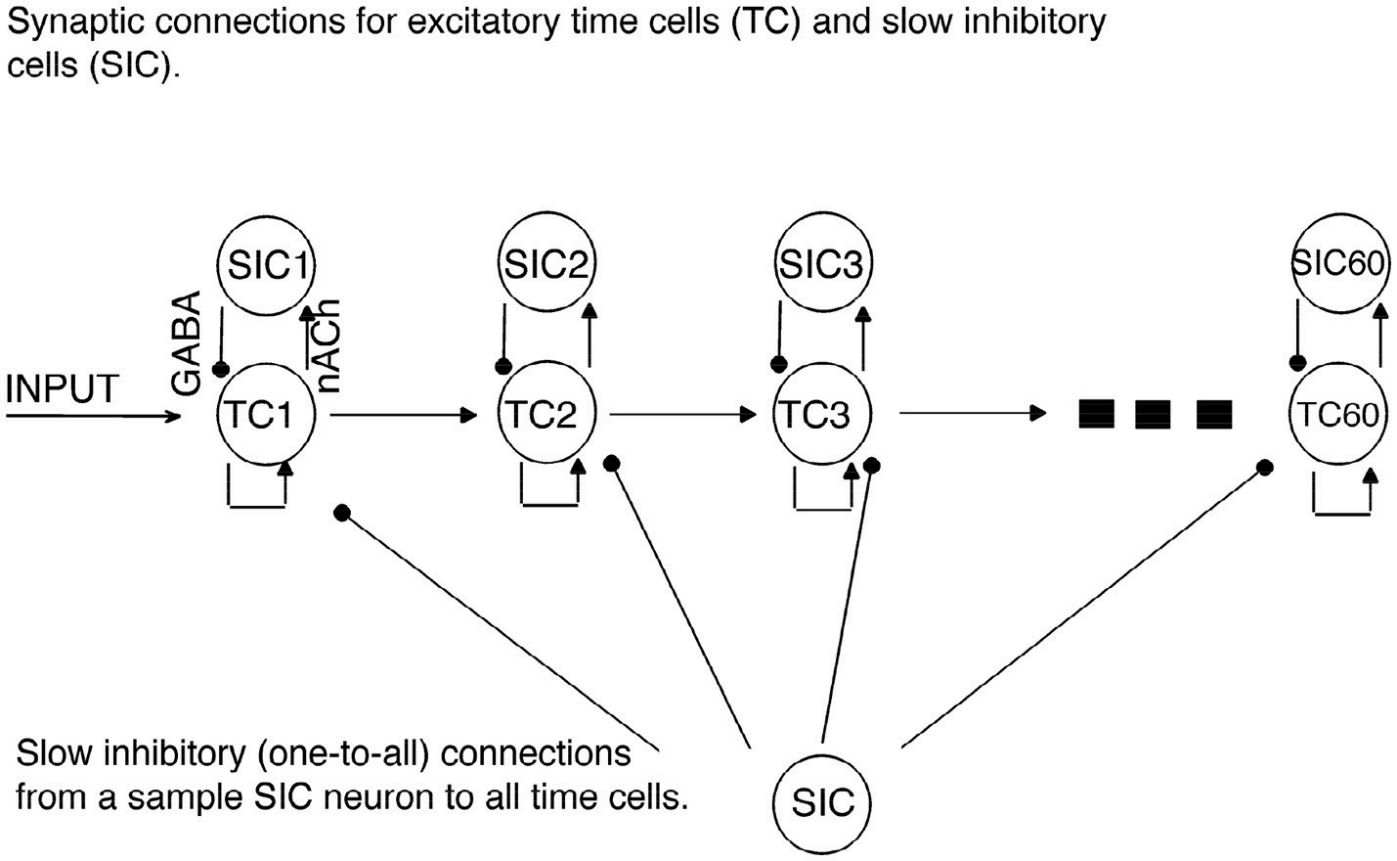
Network Architecture. Regular arrowheads denote the excitatory synaptic connections. Filled circles denote the slow inhibitory synaptic connections. Time cell (TC) ensembles are connected to each other via excitatory synaptic connections in a chain network architecture. For each time cell, we assume the existence of a slow inhibitory cell (SICn). Each time cell receives inhibition from each slow inhibitory cell.

In the current model, we use a network with feedforward connections among excitatory time cells to simulate the transmission of activity between time cells (see Figure 1). Importantly, the model focuses on the time it takes to transmit the excitatory signal from one time cell to the next time cell in the chain, namely, the inter-spike intervals (ISIs). Our assumptions regarding the role of inhibition in the model are explained below. The time cells are modeled using the spikeless integrate-and-fire type neuron model (see Ermentrout and Terman, 2010) with currents that are modeled using the Hodgkin-Huxley type formalism as follows:

$$\begin{array}{lll} C_{m}\frac{dv}{dt} & =-\left(I_{L}+I_{D}+I_{Exc}+I_{Inh}\right)\;+I_{input}+I_{noise}\; & \;\\ \; \end{array}$$

where *I*_*L*_, *I*_*D*_, *I*_*Exc*_, and *I*_*Inh*_ stand for leak, D-type potassium, excitatory and inhibitory synaptic currents, respectively. The membrane potential is reset to *v*_*R*_ = − 85*mV* when *v* = *V*_*T*_ with *V*_*T*_ = −50*mV*. *C*_*m*_ is the membrane capacitance with *C*_*m*_ = 200 μF/cm^2^. *I*_*L*_ denotes the leak current with *I*_*L*_ = *g*_*L*_(*v* − *E*_*L*_). *g*_*L*_ and *E*_*L*_ denote the leakage conductance and the reversal potential with values *g*_*L*_ = 8 μ*S* and *E*_*L*_ = − 65 *mV*. The D-type potassium current is described by the equation $I_{D}\;=g_{D}m_{d}h_{d}^{2}\left(v-E_{K}\right)$ with the maximal conductance and the reversal potential, *g*_*D*_ = 4 μ*S* and *E*_*K*_ = −90 mV, respectively (Storm, 1988; Grissmer et al., 1994). The variables describing the fast activation (*m*_*d*_) and slow inactivation (*h*_*d*_) of the D-current are described by the following differential equations: $\frac{dm_{d}}{dt}=\left(m_{d\infty }-m_{d}\right)/m_{d\tau }$, $\frac{dh_{d}}{dt}=\left(h_{d\infty }-h_{d}\right)/h_{d\tau }$ where *m*_*d∞*_ = 1 − 1/(1 + exp((*v* + 65)/2)), *m*_*dτ*_ = 0.6, *h*_*d∞*_(*v*) = 1/(1 + exp((*v* + 65))) and *h*_*dτ*_ = 1500 ms^−1^.

### 2.1. Excitatory Synaptic Currents

The synaptic excitation is given as *I*_*Exc*_ = *I*_*Exc*1_ + *I*_*Exc*2_. The current *I*_*Exc*1_ for the *n*_*th*_ time cell that represents feedforward excitatory connection from the *n* − 1_*th*_ time cell to the *n*_*th*_ time cell is given by *I*_*Exc*1_ = *g*_*Exc*_*s*(*V* − *E*_*exc*_), where *s* is the synaptic variable of the *n* − 1_*th*_ time cell. The equation describing the synaptic variable is reset to 1 with every spike of the corresponding time cell and decays exponentially with respect to the equation *ds*/*dt* = −β*s* with the decay rate β = 0.2*ms*^−1^.

The maximal excitatory conductance and the excitatory reversal potentials are given by *g*_*Exc*_ = 15 μ*S* and *E*_*Exc*_ = 0 mV, respectively. Another excitatory current, *I*_*Exc*2_ represents the recurrent excitatory connections within each time cell ensemble. The recurrent excitation is given by the equation *I*_*Exc*_ = *g*_*Excr*_*s*(*V* − *E*_*exc*_), where the maximal conductance *g*_*Excr*_ = 50μ*S* and *s* is the synaptic variable of the time cell receiving the synaptic current.

### 2.2. Model Assumptions

The model incorporates a synaptic slow inhibitory current, and this current is assumed to increase linearly with every activated time cell. The equation for the inhibitory current to a time cell is given by *I*_*Inh*_ = *Ng*_*Inh*_(*V* − *E*_*inh*_) with the maximal conductance *g*_*Inh*_ = 0.02 μ*S* and the reversal potential *E*_*Inh*_ = −100 *mV*. *N* is the number of time cells that has fired since the beginning of the current simulation. The second assumption is that the active time cell stops firing after the excitatory transmission and thus after the firing of the next time cell.

### 2.3. External Input and Initial Conditions

A 10 ms-long square pulse input current *I*_*input*_ = 4 *mA*/*cm*^2^ is given to the first time cell at the beginning of each trial. Initial values of the membrane potentials are taken to be equal to the resetting value of −75 mV. The D-current activation and inactivation variables are assumed to have initial values of *m*_*d*0_ = 0 and *h*_*d*0_ = 1, as the equilibrium value for the inactivation variable is 1 for a resting neuron. Initial values for all synaptic variables are assumed to be 0.

### 2.4. Network Architecture

A network of 60 time cells was simulated unless stated otherwise. We assumed feedforward AMPA-type excitatory connections between the time cells (Figure 1). All the time cells receive the same inhibitory current that incrementally increases in a linear fashion with the activation of each additional time cell.

### 2.5. Noise

Large network simulations were run by assigning the D-current and synaptic excitation maximal conductance values as normally distributed random variables. The standard deviation values for generating the data presented in section 3.4 are 1 for D-current maximal conductance and 5 for the maximal conductance of the synaptic excitation. In addition, the synaptic noise *I*_*noise*_ has the form $I_{noise}=\underset{n}{\sum }s_{n}\left(V-E_{exc}\right)$, where the variable *s*_*n*_ from 100 presynaptic neurons is activated at predetermined times *t*_*k*_, *k* = 1, 2, …  from a Poisson process with an average firing rate of 50 Hz (see e.g., Fourcaud and Brunel, 2002). In simulations with non-zero *I*_*noise*_, the value of the maximal conductance of the synaptic noise current is given by *g*_*noise*_ = 1 μ*S*. The variable *s*_*n*_ obeys the differential equation *ds*_*n*_/*dt* = −β_*n*_*s*_*n*_ with β_*n*_ = 0.1 *ms*^−1^.

## 3. Results

### 3.1. Time Cells *in vivo*

The characteristic feature of timing behavior is the reduced absolute timing precision with increasing target time intervals. Recent studies that aimed to find the neural correlates of the interval timing behavior in accordance with its statistical signatures discovered the so-called time cells in many different brain areas such as the striatum, hippocampus, and medial prefrontal cortex (Kraus et al., 2013; Eichenbaum, 2014; Salz et al., 2016; Tiganj et al., 2016). The conclusion that time cells can encode time came from the critical fact that different cell ensembles are activated during different time periods. Moreover, the time interval between subsequently activated cell ensembles has been observed to slowly increase with the elapsing time. In fact, it is this very property that leads to the increased absolute imprecision (constant level of relative imprecision) for the representation of longer time intervals. The increase in delays between the sequentially activated cell groups also means that “later periods” of an event are represented with fewer neurons per unit of time. This seemingly simple behavior has two neuro-computational challenges to be tackled.

Any given time interval is the combination of time intervals between the activity of consecutively firing time cells; in other words, the combination of inter-spike intervals (ISI). The sum of the ISIs determines the perceived time interval. The scalar property dictates the noise in the timing behavior or in the resultant temporal representation to increase linearly with the target time. That is, if the standard deviation of the timed responses with a mean time of 10*s* is 1*s*, then the standard deviation of the same type of responses with a mean time of 20*s* should be 2*s*. The difficulty in here is that, when the noisy data are added, the noise in the combined data decays with the square root of the total number of datum combined. For example, if we add *N* normally distributed random variables with the same mean μ and the standard deviation σ, the mean of the sum is *Nμ* and the standard deviation of the sum is √*Nσ*. On the other hand, for the scalar property to emerge, the standard deviation of the combined data should be proportional to *N*. Note that even if we add distributions with an increasing mean and standard deviation that is proportional to the mean, it takes a lot of fine-tuning to achieve the scalar property.

The main idea of the current work is that the simple exponential decay of a neuronal current can explain both the lengthening ISIs observed in time cells and the scalar variability. In our model, as in the empirical data, one time cell fires after another via chain-like excitation. We assumed that the inhibition over time cells increases along with time, making the firing of the time cells less likely during the later periods of an event. For time cells to fire within such a network setting, some other hyperpolarizing current has to decay to compensate for it. It turns out that simple exponential decay of this particular current indeed explains the lengthening ISIs and maybe, more importantly, the scalar property is manifested as an emergent property of the model. With the increasing inhibition, the hyperpolarizing intrinsic current has to decay more to account for the increased inhibition, which explains the longer ISIs. What accounts for the non-linear increase in the noise is the fact that as the inhibition increases with time, the intrinsic current has to decay more with time to compensate for the increased inhibition and the exponential decay becomes more prone to noise as small perturbations in membrane conductance now lead to larger deviations in time. In this work, we used the D-type potassium current for the mentioned hyperpolarizing intrinsic current. But there are other currents such as the A-type potassium current, which can function like the D-type potassium current (Grünewald, 2003).

### 3.2. Oscillations in One Time Cell

**Figure 2:**
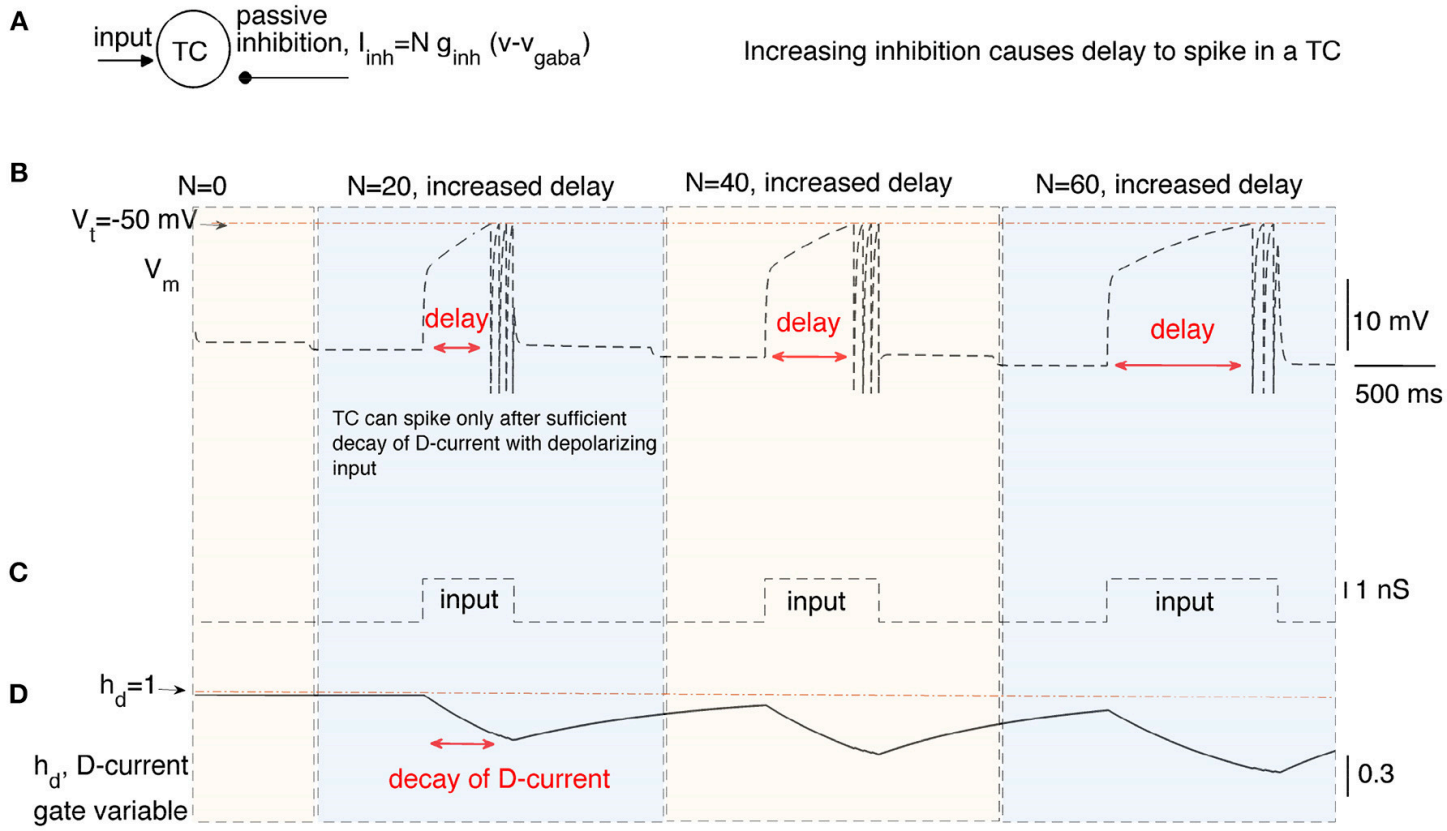
The delayed firing of a time cell (TC) in response to a square pulse input under varying inhibitory synaptic currents. **(A)** describes the inputs to the time cell, namely, input, and persistent inhibition. The coefficient *N* of the inhibitory current is increased for different time intervals to simulate the time cell under varying inhibitory inputs **(B)** with square-pulse input current **(C)**. Delay of the time cell to spike increases with the inhibition. Increasing inhibition makes the firing of the time cell less likely. Delayed spiking of the time cell is made possible by the slow inactivation of the hyperpolarizing D-current displayed in **(D)**.

In this section, we stimulate a time cell with step current stimulus for different amounts of inhibitory current to study the inhibition-dependent increase in the activation time of a time cell (see Figures 2A,B). We ran the simulations with three different levels of inhibition (i.e., *N* = 20, 40, and 60) applied for time intervals (1, 000−4, 000*ms*), (4, 000−7, 000*ms*), and (7, 000−10, 000*ms*), respectively. A square-pulse input is applied 1,000 ms after the onset of each time interval (Figure 2C).The hyperpolarizing D-current is already active at the resting potential without any applied inhibition (Figure 2D). With the application of the first external step current at *t* = 2, 000*ms* (Figure 2C), the D-current begins decreasing (Figure 2D) to compensate for the increased inhibition (*N* = 20). The time cell fires with a delay of about 600*ms*. When the inhibition coefficient is increased to *N* = 40 and *N* = 60, it takes increasingly longer for the time cell to fire (about 800 and 1, 200*ms*, respectively). This is because the hyperpolarizing D-current has to decay more to make up for the increased inhibition. Note that even though the increase in the inhibition is the same from *N* = 20 to *N* = 40 and from *N* = 40 to *N* = 60, the decay time increases non-linearly because of the exponential decay of the D-current inactivation variable (Figure 2D). When the intrinsic current is required to decay more with high inhibition during the later stages of the interval timing, the delay to spike (or ISI) becomes more prone to noise in the membrane potential. This is because the later stages of the exponential decay occur in a much slower manner and small perturbations in the membrane potential cause large deviations in decay time.

### 3.3. Larger Network Simulation

**Figure 3:**
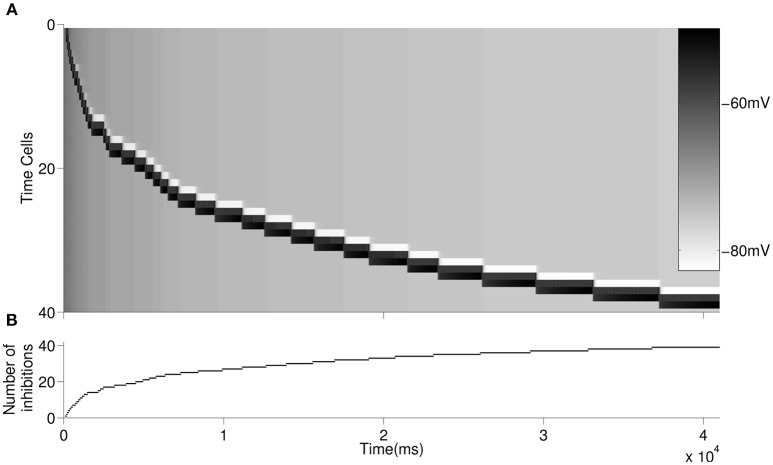
Raster plot of the larger network is displayed in **(A)**. An inhibitory cell contributes to the overall inhibition of the time cells with every activated time cell. The overall inhibition increases along with time. The increase in inhibitory synaptic current delays the firing of time cells as depicted in Figure 2. The buildup of the synaptic inhibitory current is depicted in **(B)**.

We then simulated forty time cells (see Figure 1 for the architecture) with feedforward excitatory connections to test for the increasing delay in the ISIs with elapsing time (Figure 3A). The D-current decay time constant is set to be $h_{d\tau }=3,000ms^{-1}$. In order to represent the within-ensemble excitatory connections, each time cell is designed so that it can send feedback excitation to itself (i.e., self-excitation; Figure 1). With the onset of the temporary external stimulus, the first time cell begins persistent spiking with feedback excitation and sends excitatory synaptic input to the second time cell. The activity progresses with the excitatory transmission between the time cells. Since we assume that with the activation of each time cell a slow inhibitory cell is activated persistently, the overall inhibition on the time cells increases with the progressing activity (Figure 3B). As discussed in the previous section, the increasing inhibition leads to longer delays in the activation of subsequent time cells with the elapsing time.

### 3.4. Statistical Results

**Figure 4:**
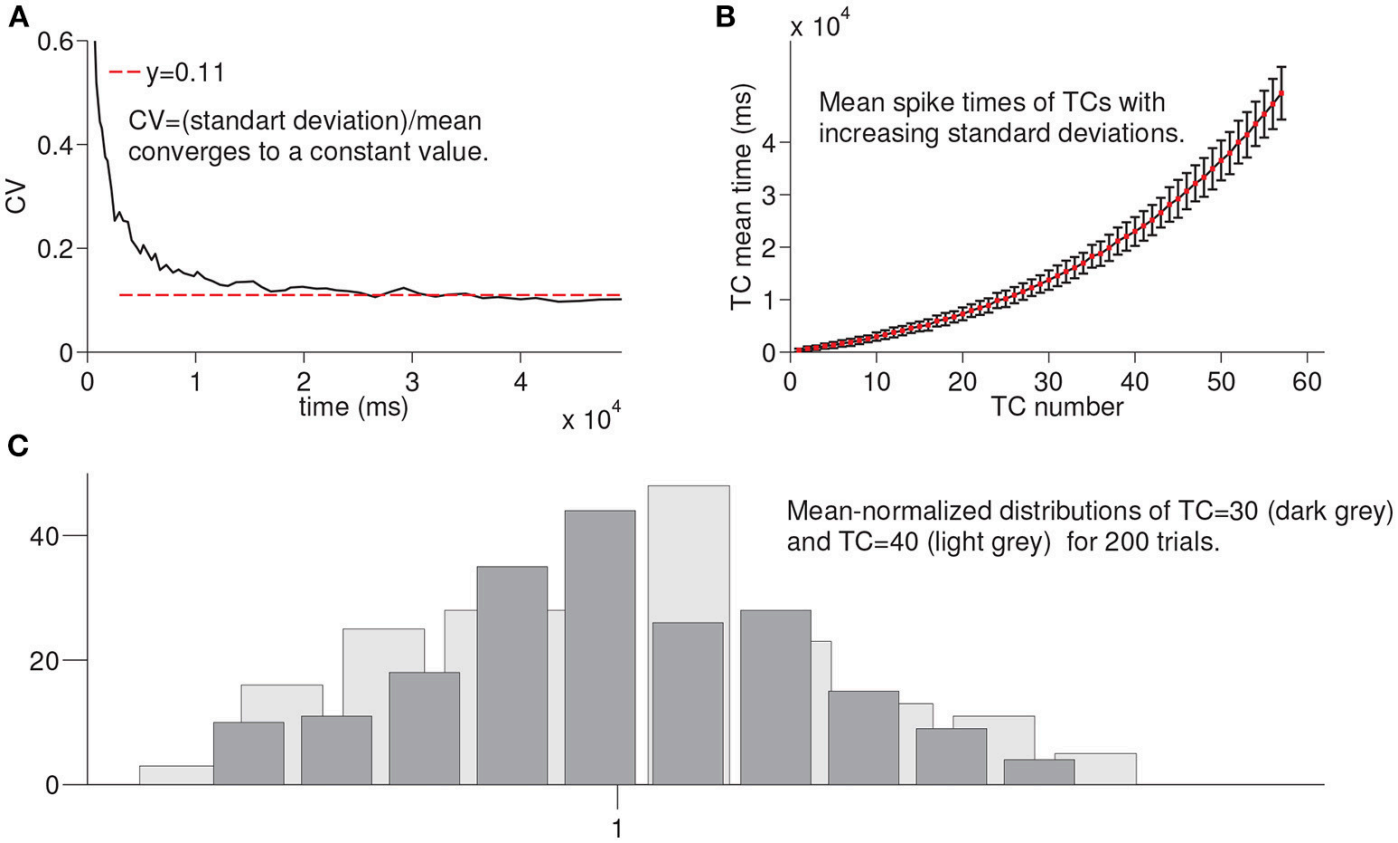
Statistical results of simulation of the larger network for every time cell for 200 trials. In order to record the firing time of each time cell, the larger network is simulated until the firing of a given time cell with noisy conductances for 200 trials. **(A)** shows the coefficient of variation of spike times of time cells with respect to the mean spike times. In **(B)**, mean spike times of each time cell are plotted with standard deviation lines. **(C)** shows the mean-normalized histograms of spike times of time cells TC 30 and TC 40, respectively.

In this section, we summarize the results of the simulations of the larger network for 200 times until the activation of each time cell is achieved to evaluate the variation in the spike times of each time cell in the chain under noisy network settings (see Methods). For example, for *TC* = 30, we run the network until the activation of the 30*th* time cell for 200 times. Figure 4A shows the coefficient of variation (CV) of spike times of the time cell with respect to the mean spike times. The red line refers to the constant value that the observed CV converges on. Figure 4B presents the mean spike times of time cells with the associated standard deviations. Note that the increase in the standard deviation is observed with the increasing cell index. Finally, Figure 4C shows the histograms of mean-normalized spike time distributions corresponding to the 30 *th* and the 40 *th* time cells. As an important property of interval timing behavior and consistent with the visual inspection of Figure 4C, the spike-time distributions that correspond to different time intervals are expected to superpose.

**Figure 5:**
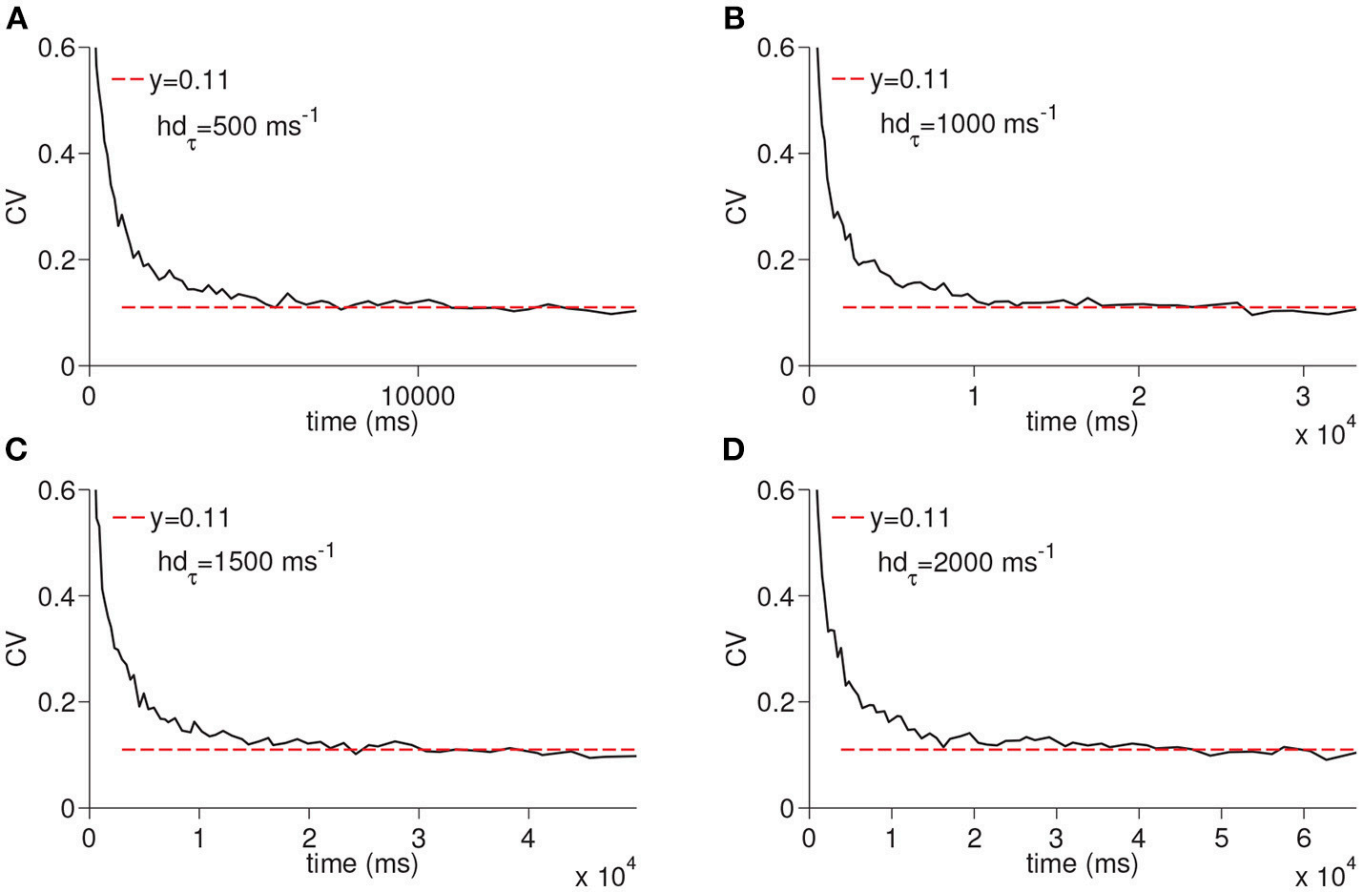
Simulation of time cell spike times for different values of the D-current inactivation variable decay rate, *h*_*dτ*_. In **(A–D)**, the values for *h*_*dτ*_ are taken to be 500, 1,000, 1,500, and 2,000 ms^−1^, respectively.

**Figure 6:**
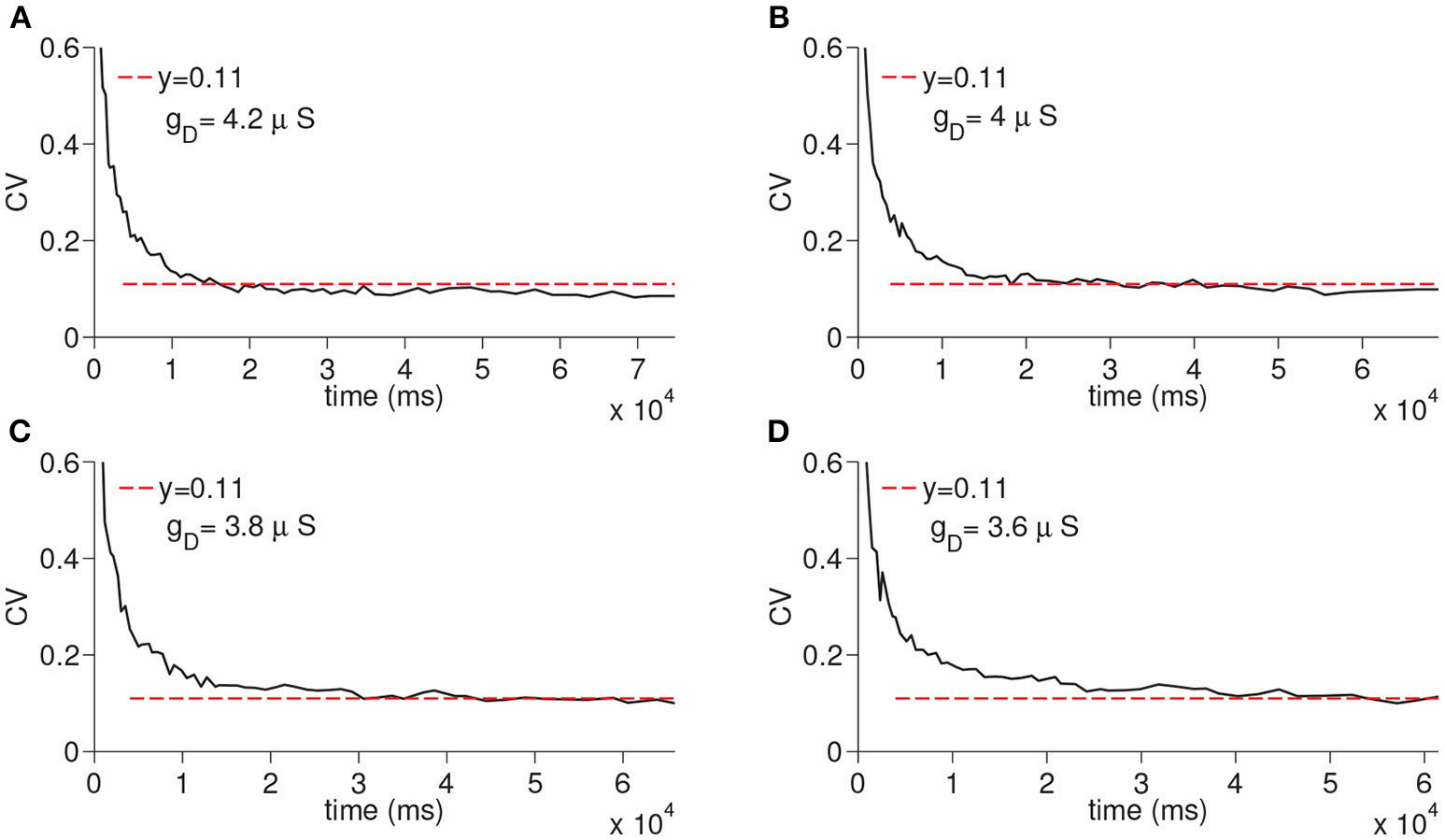
Simulation of time cell spike times for different values of the D-current maximal conductance, *g*_*D*_. In **(A–D)**, the values for *g*_*D*_ are taken to be 3.6, 3.8, 4, and 4.2 μ*S*, respectively.

We next ran the simulations to observe the dependency of CV to the D-current decay constant *h*_*dτ*_ and D-current maximal conductance *g*_*D*_ (Figures 5, 6); *h*_*dτ*_ is a key parameter as it determines the decay rate of the D-current. We simulated the large network for the increasing values of the *h*_*dτ*_ again for 200 times. The results of these simulations are displayed in Figure 5. The CV converges to the same constant value for all tested values of the D-current decay rate parameter. As expected, since D-current decays faster with the smaller values of *h*_*dτ*_, the wave speed changes considerably. The maximal D-current conductance *g*_*D*_ alters the delay to spiking. If *g*_*D*_ is small, D-current has to inactivate more to compensate for the hyperpolarizing inhibition. Hence, the smaller values of *g*_*D*_ lead to longer ISIs and higher values of *g*_*D*_ lead to shorter ISIs. When *g*_*D*_ is small, it takes less neurons to reach a given time as the ISIs are now longer. Similarly, when *g*_*D*_ is large, it takes more neurons to reach a given time since ISIs are now shorter. Adding a different number of ISIs with increased *g*_*D*_ reduces the CV as seen in Figure 6. The addition compresses the noise, hence, as *g*_*D*_ gets larger, the CV value converged on gets smaller. Importantly, the fact that the CV approaches a constant value remains intact irrespective of the changes in *g*_*D*_.

**Figure 7:**
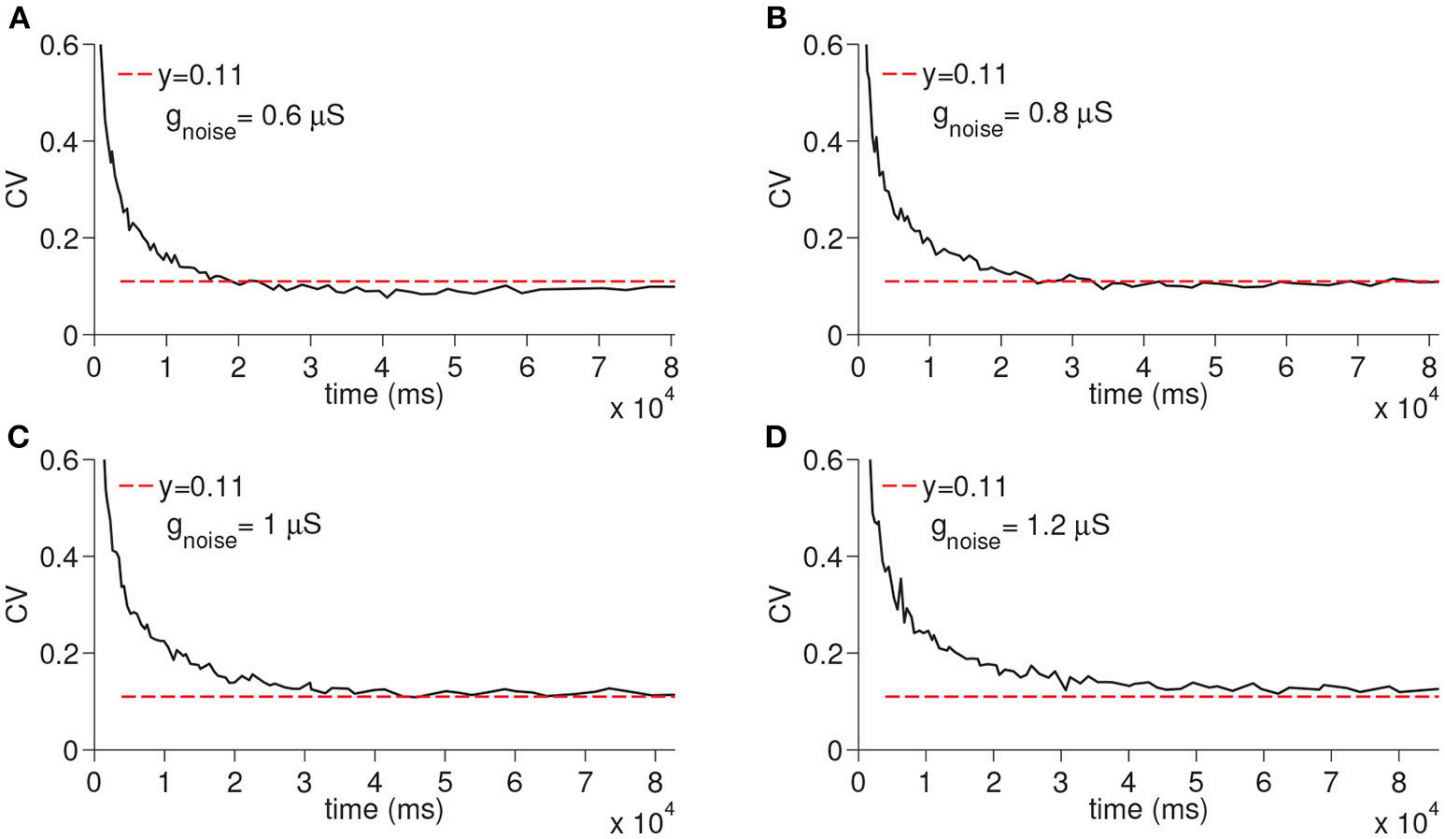
Simulation of time cell spike times for different values of the excitatory synaptic noise maximal conductance *g*_*noise*_. No other noise sources are used. In **(A–D)**, the values for *g*_*noise*_ are taken to be 0.6, 0.8, 1, and 1.2 μ*S*, respectively.

In order to evaluate the differential effect of the synaptic noise arriving from multiple presynaptic cells (*I*_*noise*_), we finally ran the simulations only with the *I*_*noise*_ removing the other sources of the noise (i.e., noise from D-current and TC-TC synaptic excitation). When the synaptic noise arriving from multiple presynaptic cells is used, the resultant CV increased depending on the maximal conductance but still remained constant again supporting the fact that virtually any manipulation that modifies the membrane potential of the time cells would result in a constant CV (see Figure 7).

## 4. Discussion

In this paper, we developed a new simple neural model of interval timing that explains the key experimental observations regarding the time cell activity patterns and is closely related to the prominent behavioral and information processing models of interval timing. The model consisted of a single layer of time cells formulated as integrate-and-fire neurons, which results in wave activity with the application of a temporary external stimulus. The observed wave activity propagates with excitatory connections between the time cells and the ISIs increase with the resultant propagating wave. The model has a simple charge-discharge, loading-unloading mechanism. The overall inhibition converging on to all the time cells charges (loads) the time cells. When a time cell begins to receive synaptic excitation, the total hyperpolarizing inhibition over that particular time cell has to be compensated by the decay of the hyperpolarizing internal current (discharge-unload). Since the inhibition increases with the total number of activated time cells, the time it takes for a given time cell to spike also increases, resulting in incrementally longer ISIs. Importantly, the simple exponential decay of the internal current determines the length of the ISI. Hence, another mechanism for the wave activity propagation that utilizes exponential decay of some process would also give similar results to those presented here. For example, each unit can activate an additional slow inhibitory current on to the next time cell. The next time cell now has to wait for the exponential decay of the slow inhibition to spike, which takes longer as the inhibition builds up with the ongoing activity.

It is well-known that inhibition can help generate many neural rhythms, such as neural synchrony, irregularity in spike-times, persistent behavior, bursting, etc. (see e.g., Whittington et al., 2000; Rotstein et al., 2005; Moustafa et al., 2008; Guo et al., 2012, 2016; Neymotin et al., 2016; Zeki and Moustafa, 2017). In the current model, inhibition is used to modulate the behavior of the time cells. In particular, with the buildup of inhibition, the delay to the firing of time cells is increased with the help of a slow intrinsic current (D-current) that activates with inhibition and inactivates with excitation.

This architecture coupled with the biophysically-plausible functional characteristics of the proposed model captures the scalar property of interval timing, namely the constant coefficient of variation of timed responses for different target durations. Due to within- and between-trial noise characteristics, when the timed behavior of the model for different target intervals is expressed on a relative timescale, the predicted timed response curves superpose (Figure 4C). Furthermore, the model is also shown to be robust with respect to different values of the key model parameters.

The fact that ISIs extend with the progressing activity alone is not enough to get scalar variability in the model behavior. In fact, we did many numerical simulations by adding normal distributions with increasing mean ISIs and standard deviation that is increasing proportionally with the mean. In these simulations, the distributions obtained by adding the normal distributions with an increasing mean and standard deviation did not result in the scalar property; in fact, a lot of fine tuning would have to be done to obtain such a linear relationship between the standard deviation and mean. This observation supports the fact that the mechanism used in our paper has scalar variability as an emergent property.

One of the important features of these findings is that interval timing behavior emerges with its well-established statistical properties based on the dynamics of the proposed architecture. The memory for time intervals is embedded within the neural circuit itself, which does not require independent encoding/decoding and comparison functions/components as proposed in some other information processing models of interval timing (e.g., Gibbon et al., 1984) (but see Matell and Meck, 2004, for a similar feature of Striatal Beat Frequency Model). Overall, the proposed model extends the scope of particularly behavioral theories of interval timing (Killeen and Fetterman, 1988; Bizo and White, 1997; Machado, 1997) in terms of their neural clock implementation based on biophysically plausible components and neuronal dynamics. In particular, the proposed model provides neural plausibility to LET-like (Machado, 1997) approaches in light of the recent neuroscientific evidence regarding time cells.

The model parameters, for example the decay rate of D-current inactivation variable or maximal excitatory-synaptic synaptic conductance, can easily alter wave speed. In Miller et al. (2006), it is shown that NMDA-type neural excitation is also capable of manipulating wave speed. To keep the model simple and parsimonious, we did not include the NMDA current in this model. However, the possible integration of other NMDA currents and/or neuromodulators such as dopamine would be a natural next step for future research. The fact that the wave speed can be easily manipulated with the model parameters gives our model the ability to account for clock speed effects that are usually interpreted in light of the Dopamine-Clock Hypothesis; dopamine agonists have been shown to lead to overestimation of the time intervals (e.g., Maricq et al., 1981; Çevik, 2003; Matell et al., 2006; Balci et al., 2008), whereas dopamine antagonist has been shown to lead to underestimation of the time intervals (e.g., Meck, 1983; Meck and Church, 1984; Drew et al., 2005).

The capacity of the model in terms of the maximum possible time interval it can measure is limited by many factors. The built-up inhibition is compensated with the exponential decay of internal current (D-current). The inactivation of the internal current puts a cap on the capacity of the model. With the given set of parameters, the model can time intervals from a few hundred milliseconds to tens of seconds, which presents an acceptable range when compared to behavioral interval timing experiments (e.g., Meck, 1983; Meck and Church, 1984; Balci et al., 2008).

Interval timing is a complex process that combines various information-processing components such as reinforcement learning (e.g., Balci et al., 2009; Balcı, 2014; Petter et al., 2018), working memory (e.g., Zeki and Moustafa, 2017), etc. We simplified our model to focus on the mechanism of the transition from one time cell ensemble to another time cell ensemble. We assumed that an inhibitory cell activates with every time cell and stays active throughout the timing episode. This type of inhibitory layer can easily be realized using CAN-type calcium current or h-type depolarizing current that is used in representing persistent activity (e.g., Zeki and Moustafa, 2017). In order to keep the model easy to follow, we limited our focus to the transmission of the signal from one time cell to another. Another assumption was that, with the activation of an excited time cell, the time cell that is already active stops firing. An adaptation current such as the slow afterhyperpolarization (AHP) can make an active time cell stop in a delayed fashion instead.

Another limitation of the proposed model is the dependence of the timing behavior on the specific connectivity pattern between the time cells. This necessitates modular circuits in the brain that have evolved to specifically keep track of time intervals in the way proposed here. A modular perspective on cognitive functions favors the possibility of such specialized timing networks. Thus, it is possible that different timing circuits (e.g., at sensory cortices, cortico-striato-thalamocortical loop, cerebellum) in essence with similar functional characteristics are present. Recent findings on time cells with sequentially dynamic activation patterns indeed strengthen this very possibility.

## Author Contributions

FB issued the question. MZ and FB designed the model idea together. MZ designed the model and run the simulations. MZ and FB wrote the article.

### Conflict of Interest Statement

The authors declare that the research was conducted in the absence of any commercial or financial relationships that could be construed as a potential conflict of interest.
